# Patient Recruitment 2.0: Become a Partner in the Patient Journey Using Digital Media

**DOI:** 10.2196/resprot.5045

**Published:** 2016-01-27

**Authors:** Michael Lindemann, Tobe Freeman, Timothy Kilchenmann, Shuree Harrison, Margaret Chan, Mark Wygonik, Lea Haines, Christian Gossens

**Affiliations:** ^1^ Roche Pharma Research and Early Development, pRED Informatics, Roche Innovation Center Basel, F. Hoffmann-La Roche Ltd Basel Switzerland; ^2^ Baden-Wuerttemberg Cooperative State University Loerrach Germany; ^3^ Pharmaceutical Research and Early Development, pRED Clinical Development, Roche Innovation Center New York, 430 East 29th Street, New York, NY 10016 New York, NY United States; ^4^ Pharmaceutical Development Clinical Operations, Roche Innovation Center Basel, F. Hoffmann-La Roche Ltd, Grenzacherstrasse 124, 4070 Basel Basel Switzerland

**Keywords:** Internet intervention, patient empowerment, cancer survivor, systematic review, web 2.0, social media

## Abstract

We describe a digital platform, Pioneering Healthcare, designed to inform and empower people who are impacted by lung cancer. The platform enables Roche to support an online conversation with patients and caregivers about lung cancer, and about the role of lung cancer clinical studies in the development of future treatment options. This conversation is live and ongoing on the platform. It provides insights about the views and motivations of patients, and about how to better support patients pursuing treatment for life-threatening illness. We discuss the strategies used to deploy Pioneering Healthcare, and the advantages of using digital platforms for raising disease awareness, increasing patient engagement and, ultimately, for boosting patient enrollment into clinical trials.

## Introduction

Establishing a digital platform that can deliver a benefit for patients and their caregivers requires insights about the severe psychological impact of receiving a diagnosis of late stage lung cancer. The impact of such a diagnosis extends beyond the patient and includes family members and caregivers. The messages and general presentation of any platform intended to support this group of individuals must acknowledge the significant sense of anxiety, grief, and loss that is experienced by individuals in such situations [1,2]. To deliver a genuine and unique benefit to visitors of our platform we must first understand these experiences, and follow best practices in the selection of informational content that we deliver to this vulnerable audience. Our ongoing research focuses on how we can support people emotionally, and with practical information, as they navigate their own personal way forward.

When detected at early stages lung cancer may be treated with curative intent using surgery, conventional chemotherapy, and radiotherapy. However, at later stages of the disease a complete remission may not be possible using standard treatment. Patients and caregivers may lose hope of finding a path toward recovery. Following a late stage lung cancer diagnosis, patients and family caregivers exhibit depression and anxiety at levels exceeding those observed in the general population [3,4]. Recent studies highlight significant benefits of psychotherapeutic intervention for this patient group as a means to ameliorate their distress, and perhaps even to improve therapeutic outcomes [5,6]. Beyond the strictly clinical setting there are benefits, and unmet needs, associated with the provision of information and psychological support for patients and caregivers [1,7-9]. Organizations such as the American Cancer Society and Cancer Research UK provide this kind of support through patient advocacy and disease awareness programs.

Digital and online activities are becoming increasingly significant in the provision of support to patients. Indeed, people confronting a serious medical diagnosis have been going online in search of emotional support and medical information for as long as the Internet has existed. The Pioneering Healthcare platform seeks to prepare and support people to make decisions about their own unique way forward as they face advanced cancer. It seeks to achieve this using an online platform that could potentially reach any patient or caregiver with access to the Internet and bring them in contact with the experiences of other people impacted by lung cancer. Accordingly, we publish a combination of basic medical information as well as content that is designed to provide visitors with psychological support and practical information [10]. We author succinct descriptions of medical aspects of the disease including information about diagnosis and treatment. We also clearly define the purpose of and the processes involved in enrolling in a clinical trial. Beyond the succinct scientific content, the platform addresses psychological and practical goals by featuring practical content about finding support services and advice about communicating with family and friends. Importantly, we enable visitors to share their insights with other visitors to the website by publishing individual messages. We also enable visitors to post comments about messages that have been contributed by other visitors.

Through the platform we seek to raise awareness of late stage lung cancer and what it means to participate in a lung cancer clinical trial. As such, basic clinical trial processes are described including eligibility and exclusion. We describe how clinical trials provide access to investigational treatments, and how this access differs from the access that patients have to standard treatment options. Finally, we provide the telephone contact information of Roche’s Trial Information Support Line based in North America. Callers can use this to reach trained health care professionals, who are able to answer questions about how to enroll in Roche-sponsored clinical trials. The site can be adapted rapidly to accommodate evolving changes to Roche’s clinical development program, such as the initiation of a new clinical trial.

**Figure 1:**
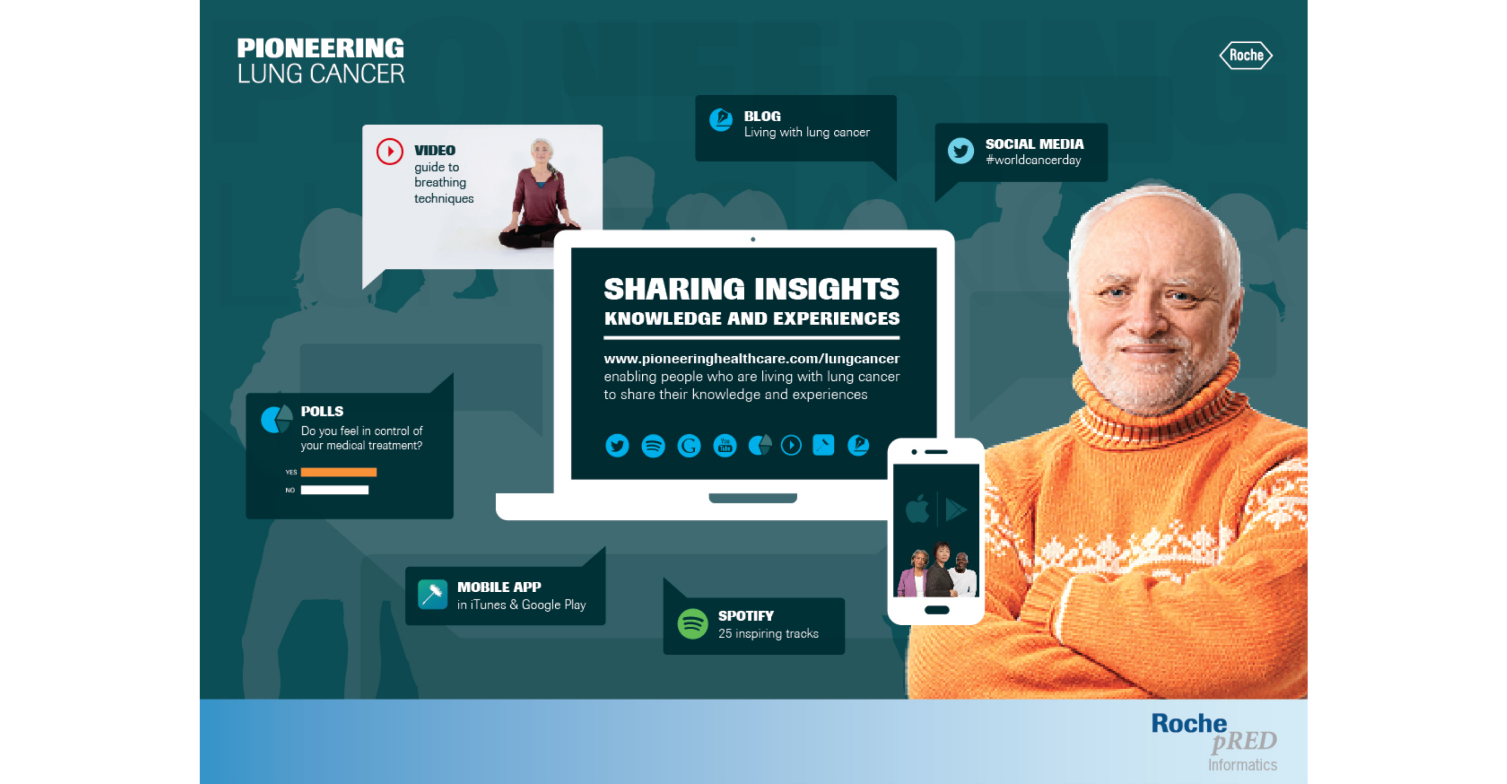
Overview of the Pioneering Healthcare platform highlighting video content, the blog, online polls, and activity on social media. Across the platform, the visual imagery is designed to communicate a sense of community and encourage visitors to engage with others online.

The platform links to additional digital resources including a mobile app and an instructional video for people recovering from thoracic surgery. These resources have been produced specifically for the platform and have been reviewed by internal and external health care professionals. Pioneering Healthcare also features a blog, a music channel on the online music streaming service Spotify, and online polls. The polls enable surveys to be conducted on topics relating to cancer treatment. Patients can contribute their views quickly and conveniently. They are then presented with the poll results to help them gauge the views of the broader online community. Various components of the platform are summarized in Figure 1.

In addition to keyword search advertising on major search engines, we promote the platform with banner messages displayed on the online social networking service Facebook. As well, we post messages about Pioneering Healthcare on the social networking service Twitter and on LinkedIn using accounts operated by F. Hoffmann-La Roche Ltd.

Visitors to the platform are able to assess their eligibility to participate in Roche-sponsored clinical trials. An online form enables visitors to enter basic details about their diagnosis and treatment history. This information is compared with the eligibility and exclusion criteria corresponding to ongoing Roche-sponsored clinical trials. The details submitted on the platform are not stored, and the online form does not capture information concerning the visitor’s identity such as their name or physical location. At the same time, the information we obtain from the form enables us to roughly estimate the proportion of website visitors who would be eligible for enrolment in specific clinical trials. Visitors who complete the form are given feedback about the likelihood that they could participate in a clinical study. The feedback page features the Trial Information Support Line telephone number to enable visitors to seek further information.

In summary, the primary goal of our platform is to inform people about the contribution of clinical trials to the development of better cancer treatment options. The platform hosts a succinct clinical summary of lung cancer and describes processes relating to clinical trial enrollment such as study inclusion and exclusion criteria and informed consent. Importantly, the platform presents the views and responses of individuals who visit the site. While this content may be considered secondary to the goal of informing people about medical and regulatory aspects of the clinical trial process, it provides important contextual information for people as they choose their own way forward following a diagnosis with cancer. To the best of our knowledge, other sponsors of cancer clinical development are not using this approach to boost awareness of clinical trials.

## Developing the Platform

The platform has been developed through an innovative collaboration between clinical operations experts and technologists at Roche. The website is based on an industry standard content management system (CMS). So-called plug-ins are used to extend the functionality of the standard CMS, including website integration with social media services and the operation of interactive online forms. A key feature of the platform is the use of digital technologies to engage in two-way interactions. The technologies enable an interaction with other website visitors, and with the operators of the platform itself. This focus on two-way interaction adds to, and complements, our goal to publish and deliver useful and emotionally supportive information to lung cancer patients. The focus on two-way interaction is aligned with the goals of Internet discussion systems characteristic of the pre-website era, such as the World Wide Web discussion system Usenet. Usenet, established in 1979, is still active today and hosts millions of digital conversations between individuals. Usenet’s enduring presence is strong confirmation of the need for two-way interaction on the Internet.

In addition to providing information about a particular topic, these systems support people seeking to engage with other people with whom they share a particular interest. Internet-based discussion systems enable people to search for, and make contact with other people. This engagement can be both empowering and rewarding for patients [11,12].

Visitors to the Pioneering Healthcare platform achieve this two-way interaction using online forms. The messages are recorded and sent automatically to Roche via email for the purposes of monitoring and content moderation before it is published on the platform. Visitors can also share links to the platform on social media services such as Facebook. The platform meets US and European regulatory requirements pertaining to patient safety. Comments and other messages received on the platform are monitored for adverse event content. If a visitor to the platform contributes a description of an adverse event then this is reported, within one business day, to the relevant regulatory compliance personnel within the organization. Those monitoring the website are trained in all relevant reporting procedures and follow industry guidelines relating to adverse event reporting.

New content is added continuously to the site in order to stimulate visitor engagement [13,14], and to guide discussion around topics that are relevant to patients and caregivers. We encourage visitors to reach out to other cancer survivors and caregivers, and to take an active role in decision-making their treatment. These messages align with recommendations published by cancer advocacy organizations such as the American Cancer Society and Cancer Research UK. The messages are also in accordance with an active literature examining the connection between information seeking behavior and positive patient perceptions of treatment and treatment outcomes [11,15].

By educating themselves and taking an active role in their care, patients become activated and more involved in their own care and treatment [15]. Self-efficacy and information seeking behavior of someone acting on their own behalf may help an individual to cope with the uncertainties of life-threatening illness [15,16]. We acknowledge that facing advanced cancer is enormously challenging. In according with the academic literature [3,6] we urge visitors to seek professional help if they experience feelings of grief and helplessness.

The resulting activity on the website is tracked and monitored systematically. We track conventional statistics such as the number of times each page is visited, as well as detailed statistics on how visitors move around the website, the pages they share on social media, and the number of messages and comments they contribute. This analysis is used to continuously refine and optimize the platform.

In developing the platform we have collaborated extensively with the legal and compliance departments at Roche. Medical content is reviewed and approved by clinicians within the organization that have specialized knowledge of relevant disease areas. Reviewers also have a sound knowledge of Roche’s ongoing clinical development plans. Collaborations with medical, legal, and compliance experts began during the initial planning phase of the platform, and these collaborations remain active now that the platform functions in an operational mode. The sustained nature of these collaborations ensures ongoing access to expert reviewers. In turn, ongoing access to reviewers ensures that we can maintain operational safety and compliance.

## Operating the Platform

The content appearing on the Pioneering Healthcare website is managed continuously, and can be modified instantaneously by a support team within Roche. The website and email server is hosted by a third party service provider offering support on a 24-hour basis 7 days a week and 365 days a year. A digital community manager is deployed to moderate interactions with visitors to the website. The community manager moderates all messages and comments before they appear live on the website. Participants of online communities have defined expectations and norms. Beyond the norms around standards of politeness and consideration, online communities demand rapid and careful management of each and every message that is sent and received on the platform. Close and continuous monitoring by the community manager enables timely and considered responses.

Each new comment or message contributed to the site is first classified according to its suitability for publication. This classification is made in terms of the content of the message, as well as the message’s emotional tone. Using email as a means to communicate with an individual contributor, the community manager may propose to publish contributions without modification, redact portions of the content, or alternatively advise the contributor politely that the comment or message is not suitable for publication. Moderation procedures ensure that published messages adhere to community standards of politeness, care, and consideration for our target audience, namely people with advanced cancer. The community manager responds to individual queries and may direct visitors to scientifically reputable online resources such as the US National Institutes of Health National Library of Medicine.

The community manager also adheres to standards of accuracy in the publication of visitor-contributed content, particularly in cases where medical and treatment themes are contributed. Contributions are reviewed and checked against specialised clinical publications maintained by F. Hoffmann-La Roche Ltd, as well as online resources maintained by the US National Library of Medicine. The community manager is also able to call upon medical and legal experts within Roche to perform this task.

We do not publish the names of individual pharmaceutical products on the platform. When a visitor includes a product name in a message or comment, the community manager seeks permission to replace the product name with the name corresponding to the pharmacological class of medications to which the product belongs. Alternatively, the community manager may propose that a specific treatment be redacted from a message or comment published on the website. This option is preferred when a visitor submits a message that makes reference to treatments that may be considered controversial by academic institutions or regulatory organizations.

The website blog is updated frequently and covers practical themes such as healthy diets for cancer survivors, advice about communicating with friends, and about services for lung cancer patients. The blog is also used to publish interviews with health care professionals and others who have expertise in the area of lung cancer treatment and survivorship. Blog posts are made to coincide with events such as the New Year and with changes in the season. These blog posts invite readers to share messages about their experiences at these emotionally important times of the year. Delivering new and topical content at such times can be an especially effective way to support patients and caregivers.

Our readers are kept up-to-date with announcements about major conferences, for example the annual American Society of Clinical Oncology (ASCO) meeting. A relatively small number of annual conferences, including ASCO, make a major contribution to the stock of new and important cancer research insights. Major conferences are, therefore, especially valuable for non-experts who seek accurate and up-to-date information. The organisers of major medical conferences routinely produce materials targeted at lay audiences. These materials include infographics showing important epidemiological statistics, lung cancer pathology, and diagrammatic presentations of a medicine’s mode of action.

Rather than duplicate the efforts made by these organisations, we seek to intermediate between patients and the organisers of these events with links to online information intended for lay audiences. The value we add is to direct our visitors to specialized, and up-to-date, information on a continuous basis. These links are carefully selected to ensure that our community is presented with information of a practical nature. As with the content we publish on the website, we distribute links to materials that offer hope and inspiration. We generally avoid materials relating to pre-clinical studies, or to otherwise speculative academic insights that are not yet available in the clinic.

The blog also provides advance notice about important patient-focused conferences, such as the annual Medicine X conference organised by Stanford University School of Medicine. Conferences such as Medicine X, and other conference platforms such as TED (Technology, Entertainment, Design) provide psychological, as well as informational, support for patients and carers. Importantly, we include advice about how to follow these events and conferences using social media. This advice takes the form of practical search tips to be used on platforms such as Twitter and Facebook, and for search engines such as Google. These tips enable visitors to find relevant content. In addition, these search tips enable our visitors to find people with shared interests and to engage with them online. It is the nature of social media that content is contributed by a large number of individuals. Individuals may contribute links to new research findings and novel insights. Others contribute by sharing this content and adding their own comments and views. Identifying suitable individuals for our visitors to engage with online requires careful research and validation. We direct our visitors to a relatively small number of experts and advocates to ensure that our visitors have a manageable list of individuals to follow and engage with.

The practical advice we provide about searching for content online is undergoing constant change and evolution. We deploy quantitative methods to track and validate the advice we provide to our visitors. By tracking online trends and publishing practical advice about online search we can reduce the effort that our visitors would otherwise need to make. Our visitors can use this information to engage online from the comfort and privacy of their home.

**Figure 2:**
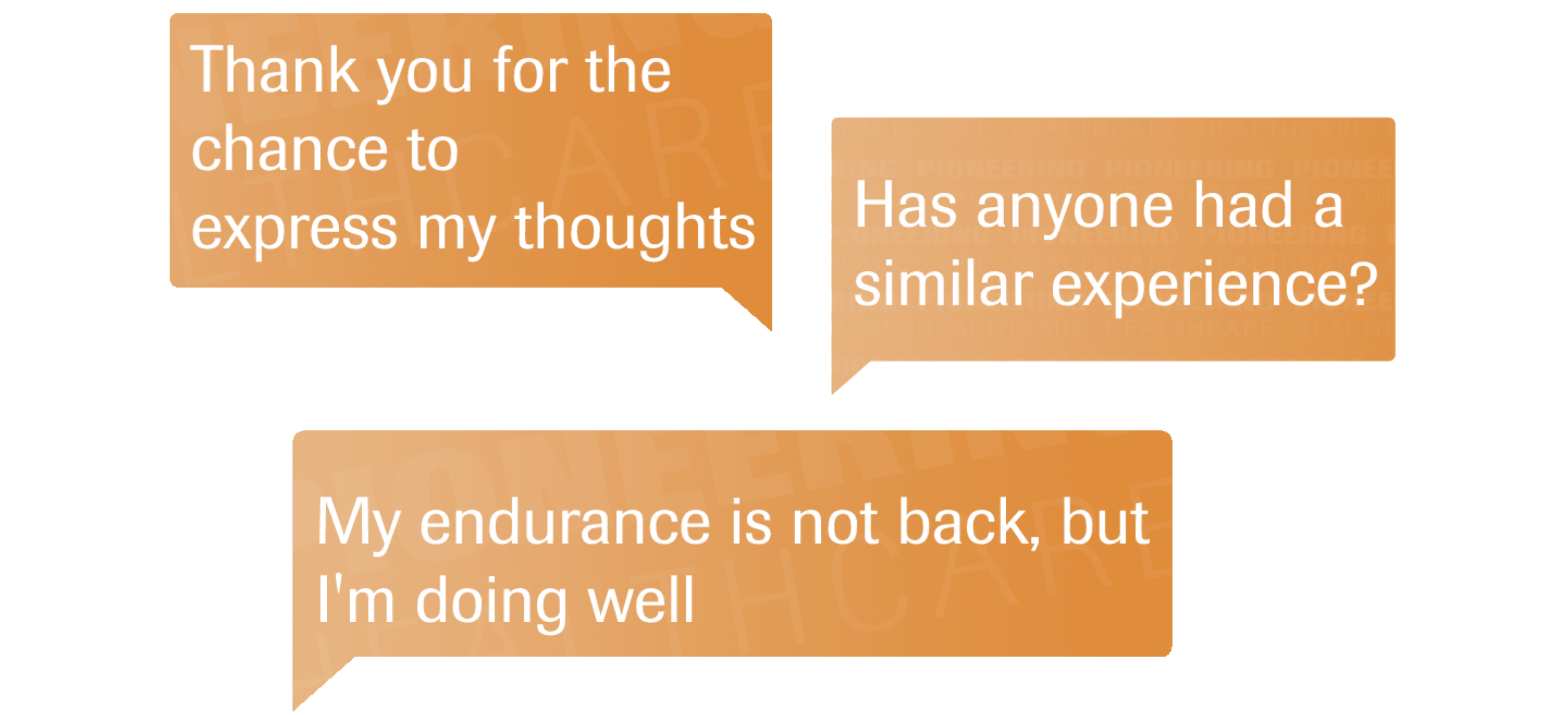
Excerpts from messages contributed by visitors to the Pioneering Healthcare platform.

Finally, the blog chronicles the online activity on our own platform. We make blog posts to highlight messages from visitors to the platform. A blog might refer to a visitor who sought further information about a particular topic. The blog post would feature links to where this information can be found online, and acknowledge and thank the visitor for bringing the query to our platform. Special blog posts are made when patient advocates visit the website and leave a comment on the platform. We focus on patient advocates who collaborate with research organisations, or non-profit organisations that sponsor research. In such blog posts we would mention the individual visitor by name, and profile the person briefly using publicly available information about the person’s advocacy activities. By mentioning the names of known patient advocates we inform our regular visitors about high profile activities within the lung cancer community. This information is of a high standard, because patient advocates who associate with research organisations and major non-profit organisations are generally vetted carefully by those organisations. The resulting content can be revisited and shared by individuals who have themselves contributed content to the platform. This in itself may be a benefit for patients and caregivers [2]. Visitors to the platform explicitly communicate their appreciation for the opportunity to publish their experiences on our platform (Figure 2).

## Discussion

We deployed the technical platform within 6 months (April 2014), including the time required to complete a review of the website content by corporate representatives from Roche’s legal and compliance departments. Rapid deployment to a live website was achieved using a standard online content management system and interactive features have been added using software plug-ins.

**Figure 3:**
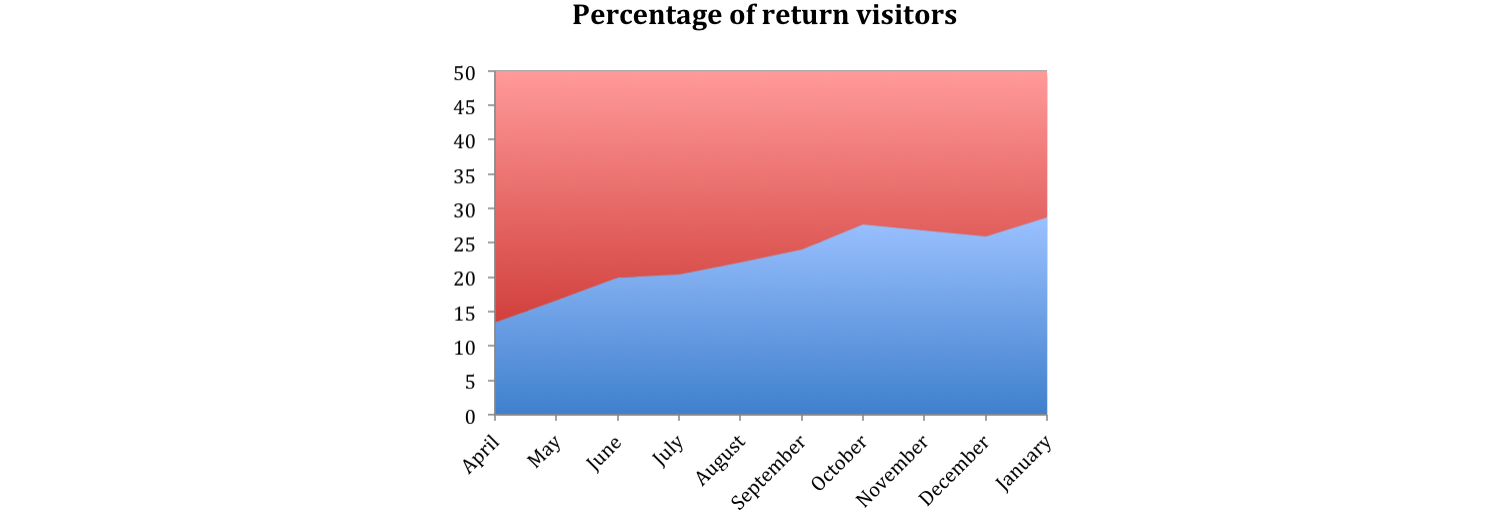
Percentage of new visitors as a percentage of existing or previous visitors. The initial 10 months of operation are shown. We observe a gradual increase in the percentage of return visitors over time.

We have enjoyed a steady flow of messages and comments from visitors to the platform, as well as a gradual increase in the proportion of return visitors to the website (Figure 3). In most cases biographical information is contributed, focusing especially on the time period from initial diagnosis and treatment. Many messages are from caregivers, including close family members. These messages from visitors to the website have proven to be highly informative in our efforts to adapt and optimize the content we publish on the Pioneering Healthcare platform. The messages we receive confirm that survivors and caregivers seek practical information about diet, their treatment options, and about dealing with feelings of isolation. The messages also confirm the sensitivities around end of life care and the need to maintain a hopeful and positive orientation.

The platform enables us to maintain an informal collaboration with patients, caregivers, and health care professionals. The collaboration is informal in the sense that visitors offer their messages voluntarily. The only restriction applying to the collaboration is that the contributions adhere to the norms of politeness and consideration for other visitors. In return, the messages that visitors contribute are published and maintained online for them. The platform provides visitors with an online presence, and contributors can share links to their messages and know that others can read their messages and respond to these messages on the platform.

The overwhelming majority of content submitted to the platform comes from patients and caregivers matching the precise diagnosis of non small-cell lung cancer that we seek to enroll in our clinical trial. This may be partly due to dominance of this patient group among lung cancer survivors [17]. It may also suggest that the targeting and promotion of the platform is effective at reaching this precise audience.

We find few people wishing to post overtly negative or even aggressive messages or critical comments about the pharmaceutical industry in general. We respond via email to those visitors and remind them of the purpose of our platform: advancing clinical research. If required, we inform the contributor politely that their content is not suitable for publication on our platform and suggest publishing their opinions on tailored discussion channels.

We use social media platforms (ie, Twitter and Facebook) to follow current trends and discussions relating to lung cancer. This digital listening activity facilitates the generation of new content for the website and enables us to follow important annual online campaigns, including lung cancer awareness month in November and World Cancer Day in February. Digital listening also enables us to have a reliable and up to date understanding of social media mores that are observed by participants in the online conversation about lung cancer. This helps us connect with our audience, and also helps to manage risks to Roche’s reputation on digital channels.

Finally, we engage with individuals using social media services such as Twitter and LinkedIn. This engagement takes the form of posts that we publish on Roche’s Twitter and LinkedIn accounts. The posts acknowledge individuals by mentioning their name, and commenting on the contribution that they make to the lives of patients and caregivers. Digital listening enables us to select these individuals carefully, avoiding risky online conversations that might damage Roche’s reputation. These activities bring us into contact with patients and health care professionals around the world.

Our platform is unique in its focus on emotional support for patients and caregivers, and in the strategy to deliver this support via an online community. A host of digital features including an interactive app, continuous content updates, and representation on social media channels enable us to deliver this support directly and on a continuous basis.

A novelty of our approach is that the platform supports visitors in their own efforts to locate and understand health information online. Practical tips published on the platform enable visitors to search for relevant online content themselves, and to connect with an extended community of people who have shared interests and who are active online.

In summary, the unique focus on emotional support and digital community is yielding authentic interactions with patients and caregivers, and boosting awareness of clinical development in the cancer field.

## Outlook

A new form of collaboration is developing between patients, health care professionals, and the sponsors of clinical trials. Guided by the need to accelerate patient recruitment and make clinical trial enrolment and retention more efficient, the sponsors of drug development are carefully listening to patients’ views and motivations.

For patients who are already enrolled in a clinical trial, this new form of collaboration is most clearly manifest in the routine inclusion of patient reported outcomes (PRO) in late stage clinical trials. Formal surveys such as the European Organization for Research and Treatment of Cancer Quality of Life Questionnaire bring a systematic quality to the acquisition and analysis of patient views.

Digital platforms may soon deliver a similar systematic quality to the acquisition and analysis of patient views. Digital platforms enable us to reach a large number of patients, but also extend our reach to caregivers, health care professionals, and other individuals who are active in patient advocacy activities. Interactive websites gather the messages and insights of a global audience, and in real time. The abundant availability of website traffic information, and other data capturing online behavior, means that this information can be analyzed with significant depth and precision.

In the creation of the platform, we have collaborated with experts in clinical operations and leveraged internal medical expertise at Roche to meet complex safety and compliance requirements. We adapt the platform continuously to ensure that it remains up to date with our current clinical development needs.

Pioneering Healthcare shows what can be achieved when clinical operations experts and technologists work together to host direct interactions with patients and caregivers. The authentic online conversation between clinical trial sponsor and patients on the Pioneering Healthcare platform deepens our insight into the needs of patients with advanced cancer.

The challenge we face is to fully understand all aspects of the insights gained from Pioneering Healthcare’s informal online collaborations with patients and caregivers. Importantly, the challenge remains to deploy these insights in the service of those impacted by advanced cancer, and in the development of the next generation of medical treatments.

## Abbreviations

ASCO: American Society of Clinical Oncology
CMS: content management system
